# Supplementary material for: Thermogenic hydrocarbon biodegradation by diverse depth-stratified microbial populations at a Scotian Basin cold seep
Source: Nat Commun. 2020 Nov 17;11:5825. doi: 10.1038/s41467-020-19648-2 (PMC7673041; doi:10.1038/s41467-020-19648-2)
Supplement: Supplementary file 1 — Supplementary Information [file 41467_2020_19648_MOESM1_ESM.pdf]

Supplementary Information for

Thermogenic hydrocarbon biodegradation by diverse depth-stratified microbial populations at a Scotian Basin cold seep

Xiyang Dong[1, 2, *], Jayne E. Rattray[2], D. Calvin Campbell[3], Jamie Webb[4], Anirban Chakraborty[2], Oyeboade Adebayo[2], Stuart Matthews[2], Carmen Li[2], Martin Fowler[4], Natasha M. Morrison[5], Adam Macdonald[5], Ryan A. Groves[2], Ian A. Lewis[2], Scott H. Wang[2], Daisuke Mayumi[6], Chris Greening[7, 8], Casey R.J. Hubert[2, *]

[1] School of Marine Sciences, Sun Yat-Sen University, Zhuhai, 519082, China

[2] Department of Biological Sciences, University of Calgary, Calgary, AB T2N 1N4, Canada

[3] Geological Survey of Canada-Atlantic, Dartmouth, NS B3B 1A6, Canada

[4] Applied Petroleum Technology (Canada), Calgary, AB T2N 1Z6, Canada

[5] Nova Scotia Department of Energy and Mines, Halifax, NS B2Y 4A2, Canada

[6] Institute for Geo-Resources and Environment, Geological Survey of Japan, National Institute of Advanced Industrial Science and Technology (AIST), 1-1-1 Higashi, Tsukuba, 305-8567, Japan

[7] School of Biological Sciences, Monash University, Clayton, VIC 3800, Australia

[8] Department of Microbiology, Biomedicine Discovery Institute, Monash University, Clayton, VIC 3800, Australia

* Corresponding authors.

Emails: dongxy23@mail.sysu.edu.cn (X.D.); chubert@ucalgary.ca (C.R.J.H.)

Supplementary Note 1

To understand the wider metabolic capabilities of these cold seep microbial communities, metabolomic analysis was combined with gene-centric analysis of quality-filtered reads, as well as genome-centric analysis of the 376 MAGs**.**

With respect to organotrophy, genes for the degradation of complex carbohydrates and peptides were prevalent. In total, ~7454 potential carbohydrate-active enzymes (CAZymes) were detected, most notably glycoside hydrolases, carbohydrate esterases, and polysaccharide lyases[1]; CAZymes were numerous in bacterial lineages such as Planctomycetes, Bacteroidetes, and Armatimonadetes **(Supplementary Data 6)**. Annotated peptidases were more evenly distributed across bacteria and archaea, with the highest proportions found in genomes assigned to Patescibacteria, *Dehalococcoidia*, Aerophobetes, and Atribacteria **(Figure 3a and Supplementary Data 7)**. Consistent with these genomic observations, metabolites associated with carbohydrate and peptide hydrolysis were detected (e.g. glucose and sucrose) **(Figure 3b)**. Many community members also possess gene sets to convert hydrolysis products to pyruvate through the Embden-Meyerhof-Parnas (185 MAGs) and Entner-Doudoroff (51 MAGs) pathways **(Figure 3b and Supplementary Data 5)**. Accordingly, both glucose 6-phosphate and pyruvate were detected in the sediment metabolomes **(Figure 3b)**. Most genomes also encoded the potential to ferment pyruvate to formate (119 MAGs), acetate (270 MAGs), lactate (14 MAGs), and ethanol (94 MAGs), as well as the potential for hydrogen production via abundant [FeFe]-hydrogenases associated with fermentation or group 3 [NiFe]-hydrogenases[2] **(Figure 3a, Supplementary Figure 11 and Supplementary Data 5)**. The ability to degrade fatty acids and other organic acids via beta-oxidation was common to Lokiarchaeota, Chloroflexi, and Proteobacteria **(Supplementary Data 8)**, with associated metabolites also detected (e.g. 6-carboxyhexanoate, 10-hydroxydecanoate, and other carboxylic acids) **(Figure 3b)**.

The most prevalent carbon fixation pathways in these sediments are the Calvin-Benson cycle (68 MAGs), Wood-Ljungdahl pathway (114 MAGs), and 4-hydroxybutyrate cycle (4 MAGs) **(Figure 3a and Supplementary Data 5)**, consistent with the abundance of

corresponding key metabolic genes **(Supplementary Figure 14)**. The sediment microbial communities include various putative sulfide, thiosulfate, ammonia, nitrite, carbon monoxide, and hydrogen oxidizers **(Supplementary Data 5)**, with provision of these compounds potentially via biological or geological processes. Canonical genes for sulfide oxidation (sulfide:quinone oxidoreductase and flavocytochrome *c* sulfide dehydrogenase) were found in 38 MAGs from diverse lineages (e.g. Proteobacteria, *Anaerolineales*, and Heimdallarchaeota) **(Supplementary Figures 3-4)**. The oxidative bacterial-type DsrAB-type dissimilatory sulfite reductases[3] were detected in four genomes from *Alpha*- and *Gammaproteobacteria* potentially responsible for sulfide oxidation **(Supplementary Figure 5)**. Five MAGs from *Epsilon*- and *Gammaproteobacteria* encoded genes for the SoxB component of the periplasmic thiosulfate-oxidizing Sox enzyme complex **(Supplementary Figure 6).** Genes for ammonia oxidation to hydroxylamine (ammonia monooxygenase) were detected in four Thaumarchaeota genomes **(Supplementary Figure 7)**, while those associated with further oxidation of nitrite (nitrite oxidoreductase) were tentatively found in five MAGs affiliated to *Anaerolineales*, Acidobacteria, Actinobacteria, and Planctomycetes **(Supplementary Figure 8)**. Gene abundance profiles suggest that these nitrogen metabolisms mainly occur in 0-20 cmbsf surface sediments **(Supplementary Figure 14)**. Three MAGs belonging to Actinobacteria, Actinobacteria, and *Alphaproteobacteria* encoded carbon monoxide dehydrogenase large subunit (CoxL), a marker for carbon monoxide oxidation **(Supplementary Figure 9)**. Ten phyla (58 MAGs) encoded group 1 and group 2 [NiFe]-hydrogenases associated with hydrogenotrophic respiration, with certain lineages, including *Deltaproteobacteria*, Planctomycetes, *Dehalococcoidia*, Actinobacteria, and Aerophobetes, encoding subgroups (1a, 1b) that support hydrogenotrophic sulfate reduction and halorespiration[4, 5] **(Supplementary Figure 10)**. Additionally, 197 MAGs from 35 phyla encode cofactor-coupled group 3 [NiFe]-hydrogenases (**Supplementary Figure 11)** and energy-converting group 4 [NiFe]-hydrogenases (**Supplementary Figure 12)**, both of which are known to be physiologically reversible[2].

Metabolomics revealed organohalides as potential respiratory electron acceptors in the sediments, e.g. dichlorotoluene and dichlorodiphenyldichloroethane **(Figure 3b)**.

Accordingly, reductive dehalogenases were encoded in 54 MAGs from 13 phyla, including lineages such as Chloroflexi, Lokiarchaeota, Bathyarchaeota, and Proteobacteria **(Supplementary Figure 13)**. Organisms encoding these enzymes may conserve energy by coupling oxidation of H_2 and other electron donors to the reduction of organochloride compounds like the ones detected in the sediments. Full gene sets for dissimilatory reduction of sulfate to sulfide (*sat*, *aprAB* and *dsrAB*) were found in 12 genomes from *Deltaproteobacteria* and Zixibacteria **(Supplementary Figure 5)**. Thirty bacterial members of these sediment communities are predicted to use nitrate or nitrite as electron acceptors, though none encoded all of the genes necessary for complete denitrification or dissimilatory nitrate reduction to ammonia (**Supplementary Data 5)**. Terminal oxidases were also detected, but in line with oxygen only permeating the upper centimeters of marine sediments, these genes were mainly found in lineages such as Proteobacteria and Thaumarchaeota that dominated the upper sediment layers **(Supplementary Figure 14)**.

Supplementary Table 1 Extractable organic matter compositions of four sediment subsamples.

Depth (cmbsf)		53-60	208-213	227-232	310-315
TOC (%)		0.25	0.35	0.36	0.56
EOM (mg/kg rock)		104	361	177	168
wt% of EOM	SAT	47.5	52.1	35.0	24.6
	ARO	10.0	11.8	13.9	12.5
	POL	42.5	26.5	17.9	36.4
	ASP	0.0	9.6	33.2	26.4
δ^{13}C-Sat (‰)		ND	-31.5	ND	ND
δ^{13}C-Aro (‰)		ND	-29.6	ND	ND

Abbreviations: TOC, total organic carbon; EOM, extractable organic matter; SAT, saturated hydrocarbons; ARO, aromatic hydrocarbons; POL, polars; ASP, asphaltenes; wt%, weight percentage; ND, not determined.

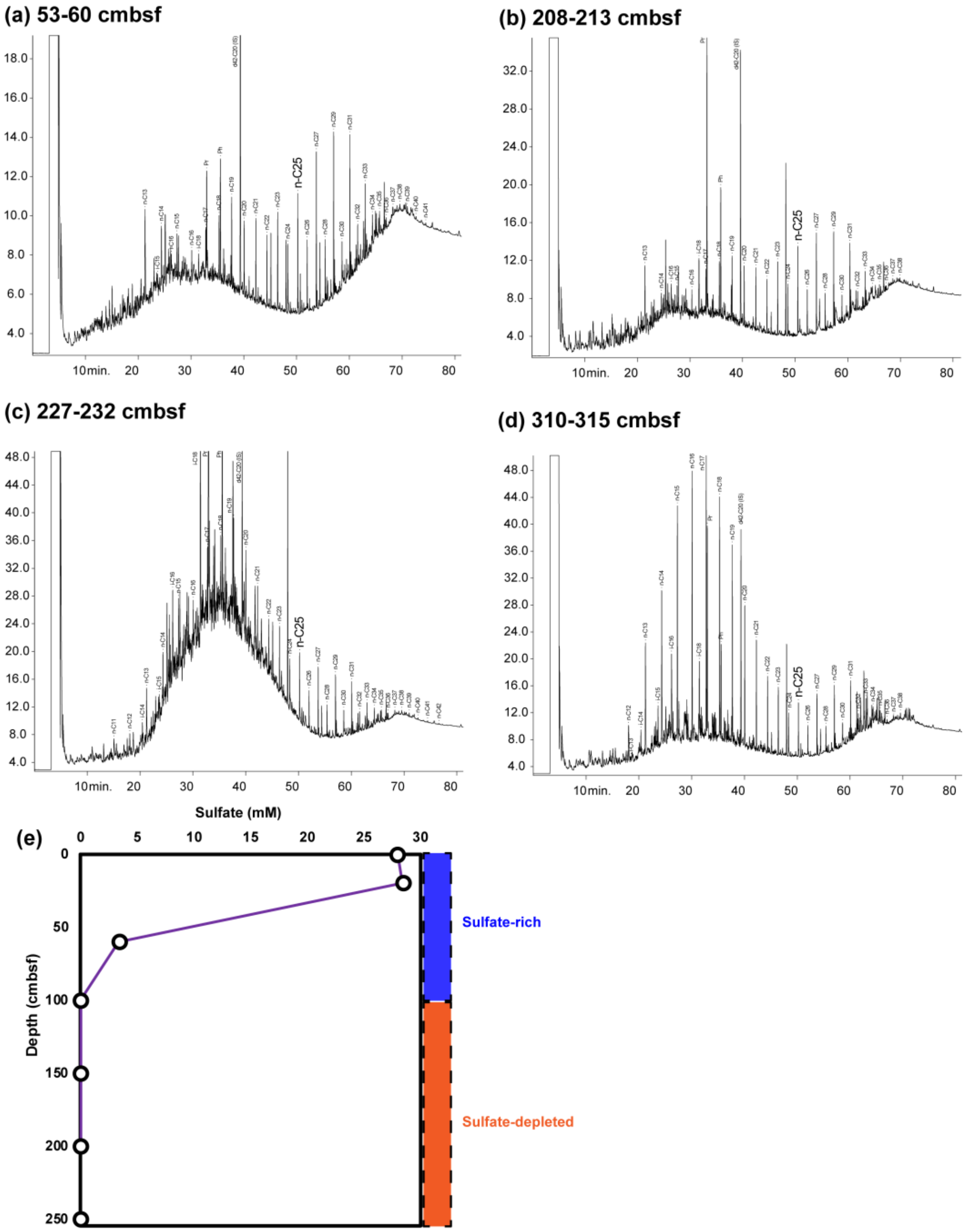

Supplementary Figure 1 Extractable organic matter from subsampled sediments and sulfate concentrations from porewater. GC-FID chromatograms showing unresolved complex mixture (UCM) humps for four different depths including (a) 53-60 cmbsf, (b) 208-213 cmbsf, (c) 227-232 cmbsf, and (d) 310-315 cmbsf; (e) porewater concentrations of sulfate along depths. For (a)-(d), y-axis: detector response; x-axis: retention time (minutes). Additional parameters for the extractable organic matter of these samples are provided in **Supplementary Table 1**.

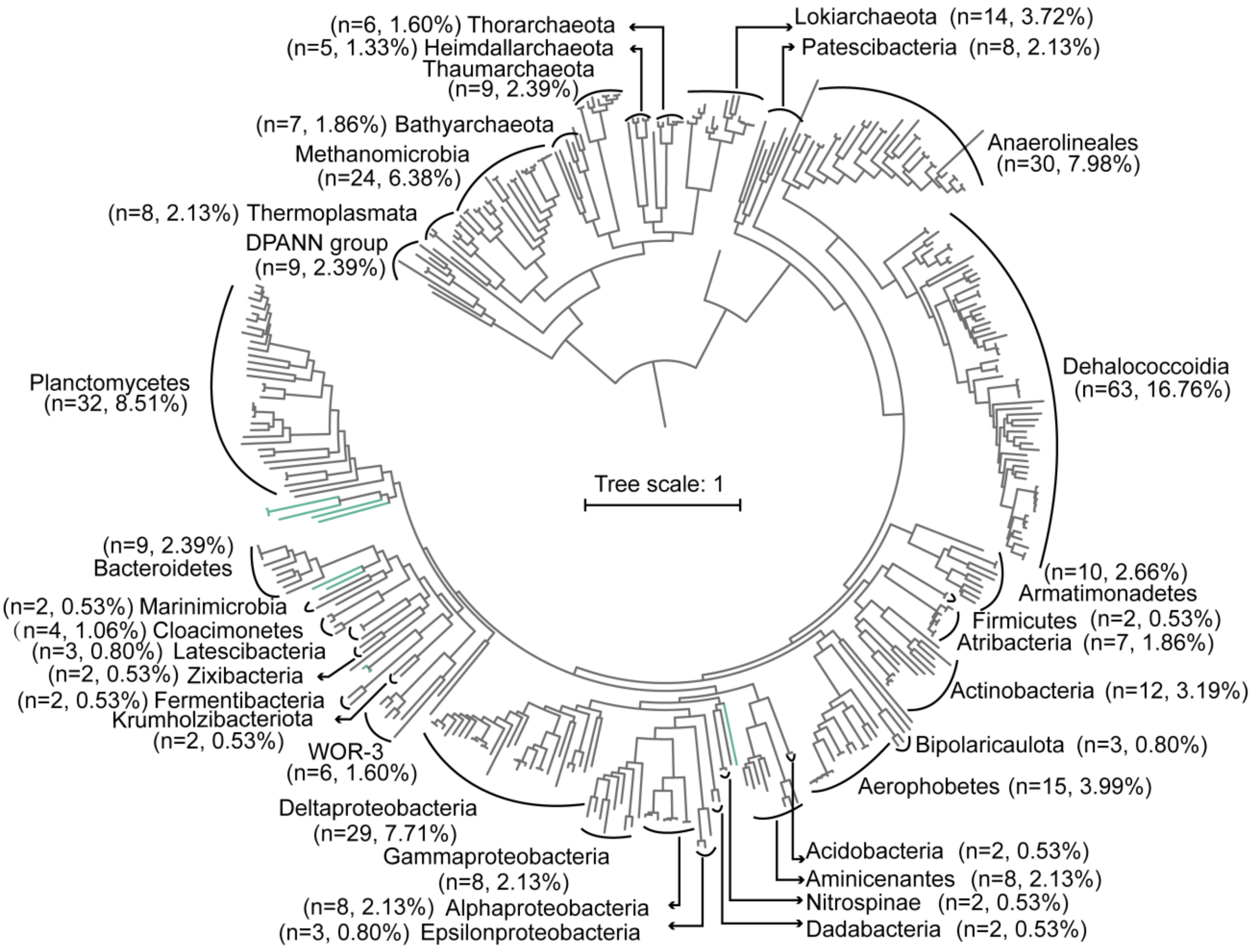

Supplementary Figure 2 Phylogenetic placement of 376 reconstructed metagenome-assembled genomes. A maximum-likelihood phylogenomic tree was built based on concatenated amino acid sequences of 43 conserved single-copy genes using RAxML with the PROTGAMMALG model. The scale bar represents 1 amino acid substitution per sequence position. Numbers in parentheses show total MAGs recovered. Phyla with only one detected MAG are not labelled. Ten MAGs corresponded to unclassified phylum-level lineages (green branches; unlabelled). The full tree file with references is available in **Supplementary Data 4**.

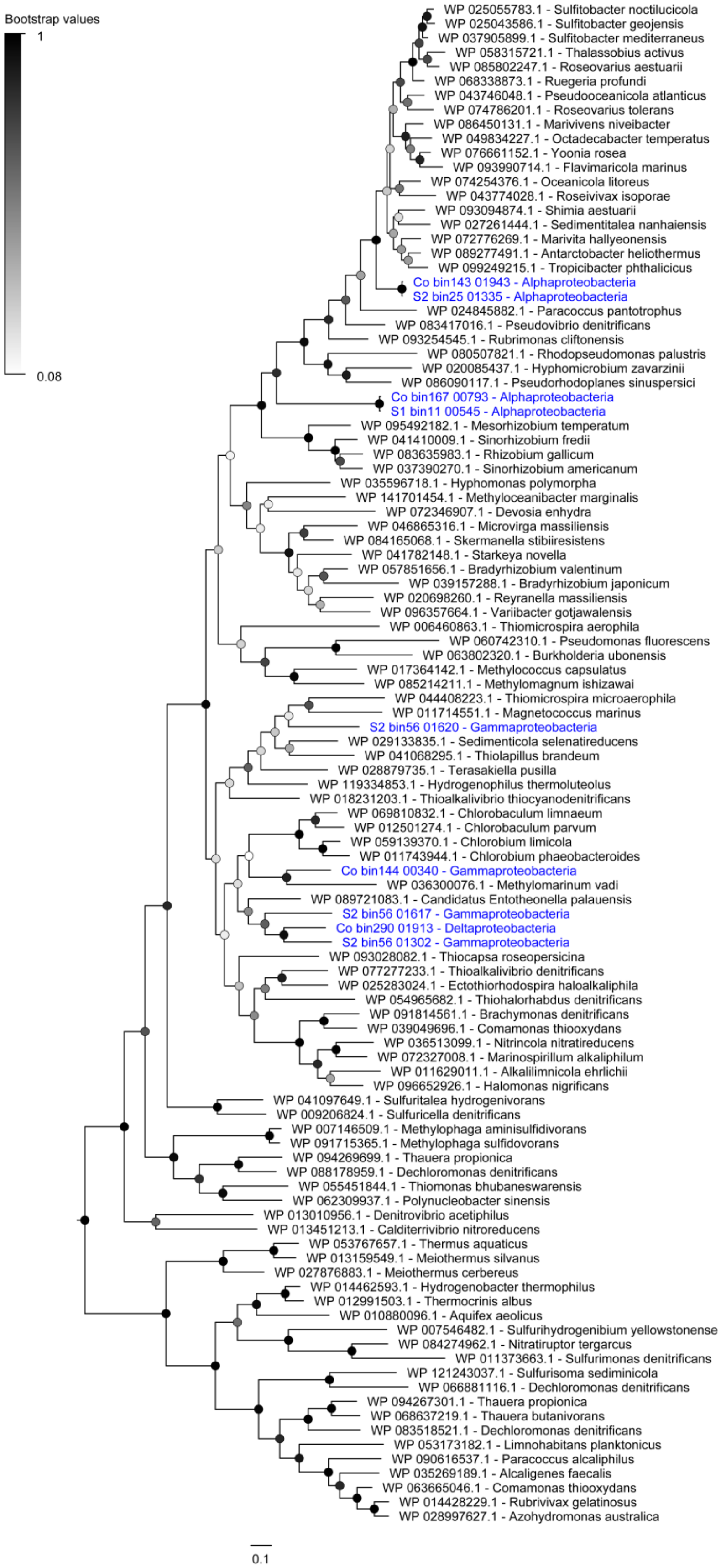

Bootstrap values
1
0.08
WP 025055783.1 - Sulfitobacter noctilucicola
WP 025043586.1 - Sulfitobacter geojensis
WP 037905899.1 - Sulfitobacter mediterraneus
WP 058315721.1 - Thalassobius activus
WP 085802247.1 - Roseovarius aestuarii
WP 068338873.1 - Ruegeria profundi
WP 043746048.1 - Pseudooceanicola atlanticus
WP 074786201.1 - Roseovarius tolerans
WP 086450131.1 - Marivivens niveibacter
WP 049834227.1 - Octadecabacter temperatus
WP 076661152.1 - Yoonia rosea
WP 093990714.1 - Flavimaricola marinus
WP 074254376.1 - Oceanicola litoreus
WP 043774028.1 - Roseivivax isoporae
WP 093094874.1 - Shimia aestuarii
WP 027261444.1 - Sedimentitalea nanhaiensis
WP 072776269.1 - Marivita hallyeonensis
WP 089277491.1 - Antarctobacter heliothermus
WP 099249215.1 - Tropicibacter phthalicicus
Co bin143 01943 - Alphaproteobacteria
S2 bin25 01335 - Alphaproteobacteria
WP 024845882.1 - Paracoccus pantotrophus
WP 083417016.1 - Pseudovibrio denitrificans
WP 093254545.1 - Rubrimonas cliftonensis
WP 080507821.1 - Rhodopseudomonas palustris
WP 020085437.1 - Hyphomicrobium zavarzinii
WP 086090117.1 - Pseudorhodoplanes sinuspersici
Co bin167 00793 - Alphaproteobacteria
S1 bin11 00545 - Alphaproteobacteria
WP 095492182.1 - Mesorhizobium temperatum
WP 041410009.1 - Sinorhizobium fredii
WP 083635983.1 - Rhizobium gallicum
WP 037390270.1 - Sinorhizobium americanum
WP 035596718.1 - Hyphomonas polymorpha
WP 141701454.1 - Methyloceanibacter marginalis
WP 072346907.1 - Devosia enhydra
WP 046865316.1 - Microvirga massiliensis
WP 084165068.1 - Skermanella stibiiresistens
WP 041782148.1 - Starkeya novella
WP 057851656.1 - Bradyrhizobium valentinum
WP 039157288.1 - Bradyrhizobium japonicum
WP 020698260.1 - Reyranella massiliensis
WP 096357664.1 - Variibacter gotjawalensis
WP 006460863.1 - Thiomicrospira aerophila
WP 060742310.1 - Pseudomonas fluorescens
WP 063802320.1 - Burkholderia ubonensis
WP 017364142.1 - Methylococcus capsulatus
WP 085214211.1 - Methylomagnum ishizawai
WP 044408223.1 - Thiomicrospira microaerophila
WP 011714551.1 - Magnetococcus marinus
S2 bin56 01620 - Gammaproteobacteria
WP 029133835.1 - Sedimenticola selenatireducens
WP 041068295.1 - Thiolapillus brandeum
WP 028879735.1 - Terasakiella pusilla
WP 119334853.1 - Hydrogenophilus thermoluteolus
WP 018231203.1 - Thioalkalivibrio thiocyanodenitrificans
WP 069810832.1 - Chlorobaculum limnaeum
WP 012501274.1 - Chlorobaculum parvum
WP 059139370.1 - Chlorobium limicola
WP 011743944.1 - Chlorobium phaeobacteroides
Co bin144 00340 - Gammaproteobacteria
WP 036300076.1 - Methylomarinum vadi
WP 089721083.1 - Candidatus Entotheonella palauensis
S2 bin56 01617 - Gammaproteobacteria
Co bin290 01913 - Deltaproteobacteria
S2 bin56 01302 - Gammaproteobacteria
WP 093028082.1 - Thiocapsa roseopersicina
WP 077277233.1 - Thioalkalivibrio denitrificans
WP 025283024.1 - Ectothiorhodospira haloalkaliphila
WP 054965682.1 - Thiohalorhabdus denitrificans
WP 091814561.1 - Brachymonas denitrificans
WP 039049696.1 - Comamonas thiooxydans
WP 036513099.1 - Nitrincola nitratireducens
WP 072327008.1 - Marinospirillum alkaliphilum
WP 011629011.1 - Alkalilimnicola ehrlichii
WP 096652926.1 - Halomonas nigrificans
WP 041097649.1 - Sulfuritalea hydrogenivorans
WP 009206824.1 - Sulfuricella denitrificans
WP 007146509.1 - Methylophaga aminisulfidivorans
WP 091715365.1 - Methylophaga sulfidovorans
WP 094269699.1 - Thauera propionica
WP 088178959.1 - Dechloromonas denitrificans
WP 055451844.1 - Thiomonas bhubaneswarensis
WP 062309937.1 - Polynucleobacter sinensis
WP 013010956.1 - Denitrovibrio acetiphilus
WP 013451213.1 - Calditerrivibrio nitroreducens
WP 053767657.1 - Thermus aquaticus
WP 013159549.1 - Meiothermus silvanus
WP 027876883.1 - Meiothermus cerbereus
WP 014462593.1 - Hydrogenobacter thermophilus
WP 012991503.1 - Thermocrinis albus
WP 010880096.1 - Aquifex aeolicus
WP 007546482.1 - Sulfurihydrogenibium yellowstonense
WP 084274962.1 - Nitratiruptor tergarcus
WP 011373663.1 - Sulfurimonas denitrificans
WP 121243037.1 - Sulfurisoma sediminicola
WP 066881116.1 - Dechloromonas denitrificans
WP 094267301.1 - Thauera propionica
WP 068637219.1 - Thauera butanivorans
WP 083518521.1 - Dechloromonas denitrificans
WP 053173182.1 - Limnohabitans planktonicus
WP 090616537.1 - Paracoccus alcaliphilus
WP 035269189.1 - Alcaligenes faecalis
WP 063665046.1 - Comamonas thiooxydans
WP 014428229.1 - Rubrivivax gelatinosus
WP 028997627.1 - Azohydromonas australica
0.1

Supplementary Figure 3 Maximum-likelihood tree of amino acid sequences of flavocytochrome *c* sulfide dehydrogenase (FCC), a marker for sulfide oxidation. The tree shows sequences from cold seep metagenome-assembled genomes obtained in this study (blue) alongside representative reference sequences (black). The tree was constructed using the JTT matrix-based model, used all sites, and was bootstrapped with 50 replicates and midpoint-rooted.

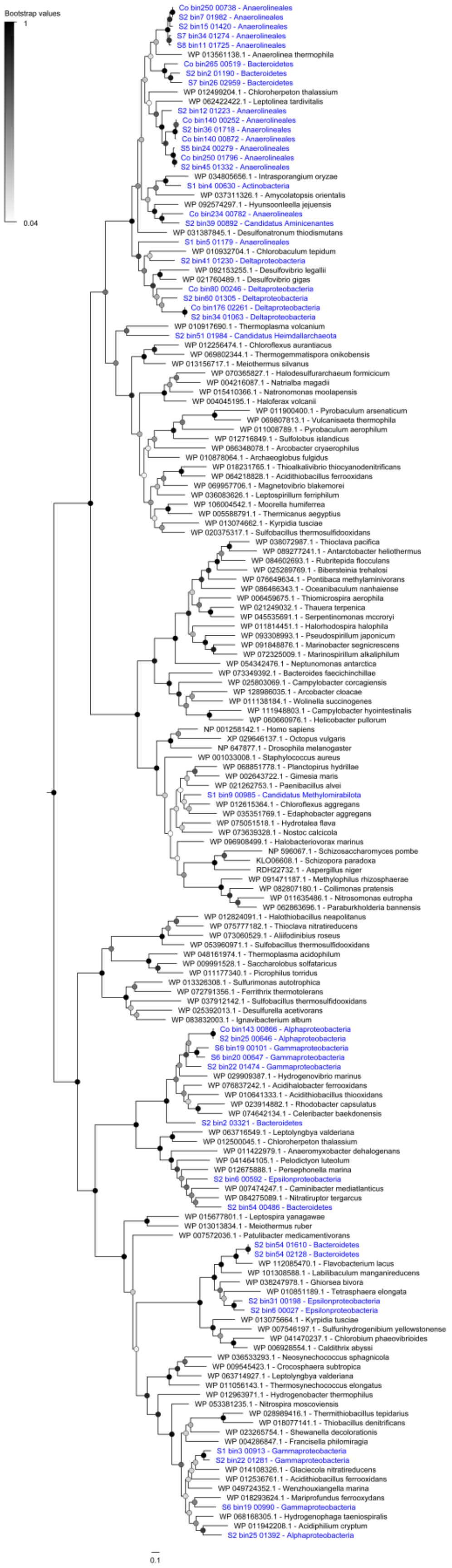
Bootstrap values
1
0.04
Co bin250 00738 - Anaerolineales
S2 bin7 01982 - Anaerolineales
S2 bin15 01420 - Anaerolineales
S7 bin34 01274 - Anaerolineales
S8 bin11 01725 - Anaerolineales
WP 013561138.1 - Anaerolinea thermophila
Co bin265 00519 - Bacteroidetes
S2 bin2 01190 - Bacteroidetes
S7 bin26 02959 - Bacteroidetes
WP 012499204.1 - Chloroherpeton thalassium
WP 062422422.1 - Leptolinea tardivitalis
S2 bin12 01223 - Anaerolineales
Co bin140 00252 - Anaerolineales
S2 bin36 01718 - Anaerolineales
Co bin140 00872 - Anaerolineales
S5 bin24 00279 - Anaerolineales
Co bin250 01796 - Anaerolineales
S2 bin45 01332 - Anaerolineales
WP 034805656.1 - Intrasporangium oryzae
S1 bin4 00630 - Actinobacteria
WP 037311326.1 - Amycolatopsis orientalis
WP 092574297.1 - Hyunsoonleella jejuensis
Co bin234 00782 - Anaerolineales
S2 bin39 00892 - Candidatus Aminicenantes
WP 031387845.1 - Desulfonatronum thiodismutans
S1 bin5 01179 - Anaerolineales
WP 010932704.1 - Chlorobaculum tepidum
S2 bin41 01230 - Deltaproteobacteria
WP 092153255.1 - Desulfovibrio legallii
WP 021760489.1 - Desulfovibrio gigas
Co bin80 00246 - Deltaproteobacteria
S2 bin60 01305 - Deltaproteobacteria
Co bin176 02261 - Deltaproteobacteria
S2 bin34 01063 - Deltaproteobacteria
WP 010917690.1 - Thermoplasma volcanium
S2 bin51 01984 - Candidatus Heimdallarchaeota
WP 012256474.1 - Chloroflexus aurantiacus
WP 069802344.1 - Thermogemmatispora onikobensis
WP 013156717.1 - Meiothermus silvanus
WP 070365827.1 - Halodesulfurarchaeum formicicum
WP 004216087.1 - Natrialba magadii
WP 015410366.1 - Natronomonas moolapensis
WP 004045195.1 - Haloferax volcanii
WP 011900400.1 - Pyrobaculum arsenaticum
WP 069807813.1 - Vulcanisaeta thermophila
WP 011008789.1 - Pyrobaculum aerophilum
WP 012716849.1 - Sulfolobus islandicus
WP 066348078.1 - Arcobacter cryaerophilus
WP 010878064.1 - Archaeoglobus fulgidus
WP 018231765.1 - Thioalkalivibrio thiocyanodenitrificans
WP 064218828.1 - Acidithiobacillus ferrooxidans
WP 069957706.1 - Magnetovibrio blakemorei
WP 036083626.1 - Leptospirillum ferriphilum
WP 106004542.1 - Moorella humiferrea
WP 005588791.1 - Thermicanus aegyptius
WP 013074662.1 - Kyrpidia tusciae
WP 020375317.1 - Sulfobacillus thermosulfidooxidans
WP 038072987.1 - Thioclava pacifica
WP 089277241.1 - Antarctobacter heliothermus
WP 084602693.1 - Rubritepida flocculans
WP 025289769.1 - Bibersteinia trehalosi
WP 076649634.1 - Pontibaca methylaminivorans
WP 086466343.1 - Oceanibaculum nanhaiense
WP 006459675.1 - Thiomicrospira aerophila
WP 021249032.1 - Thauera terpenica
WP 045535691.1 - Serpentinomonas mccroryi
WP 011814451.1 - Halorhodospira halophila
WP 093308993.1 - Pseudospirillum japonicum
WP 091848876.1 - Marinobacter segnicrescens
WP 072325009.1 - Marinospirillum alkaliphilum
WP 054342476.1 - Neptunomonas antarctica
WP 073349392.1 - Bacteroides faecichinchillae
WP 025803069.1 - Campylobacter corcagiensis
WP 128986035.1 - Arcobacter cloacae
WP 011138184.1 - Wolinella succinogenes
WP 111948803.1 - Campylobacter hyointestinalis
WP 060660976.1 - Helicobacter pullorum
NP 001258142.1 - Homo sapiens
XP 029646137.1 - Octopus vulgaris
NP 647877.1 - Drosophila melanogaster
WP 001033008.1 - Staphylococcus aureus
WP 068851778.1 - Planctopirus hydrillae
WP 002643722.1 - Gimesia maris
WP 021262753.1 - Paenibacillus alvei
S1 bin9 00985 - Candidatus Methylomirabilota
WP 012615364.1 - Chloroflexus aggregans
WP 035351769.1 - Edaphobacter aggregans
WP 075051518.1 - Hydrotalea flava
WP 073639328.1 - Nostoc calcicola
WP 096908499.1 - Halobacteriovorax marinus
NP 596067.1 - Schizosaccharomyces pombe
KLO06608.1 - Schizopora paradoxa
RDH22732.1 - Aspergillus niger
WP 091471187.1 - Methylophilus rhizosphaerae
WP 082807180.1 - Collimonas pratensis
WP 011635486.1 - Nitrosomonas eutropha
WP 062863696.1 - Paraburkholderia bannensis
WP 012824091.1 - Halothiobacillus neapolitanus
WP 075777182.1 - Thioclava nitratireducens
WP 073060529.1 - Aliifodinibius roseus
WP 053960971.1 - Sulfobacillus thermosulfidooxidans
WP 048161974.1 - Thermoplasma acidophilum
WP 009991528.1 - Saccharolobus solfataricus
WP 011177340.1 - Picrophilus torridus
WP 013326308.1 - Sulfurimonas autotrophica
WP 072791356.1 - Ferrithrix thermotolerans
WP 037912142.1 - Sulfobacillus thermosulfidooxidans
WP 025392013.1 - Desulfurella acetivorans
WP 083832003.1 - Ignavibacterium album
Co bin143 00866 - Alphaproteobacteria
S2 bin25 00646 - Alphaproteobacteria
S6 bin19 00101 - Gammaproteobacteria
S6 bin20 00647 - Gammaproteobacteria
S2 bin22 01474 - Gammaproteobacteria
WP 029909387.1 - Hydrogenovibrio marinus
WP 076837242.1 - Acidihalobacter ferrooxidans
WP 010641333.1 - Acidithiobacillus thiooxidans
WP 023914882.1 - Rhodobacter capsulatus
WP 074642134.1 - Celeribacter baekdonensis
S2 bin2 03321 - Bacteroidetes
WP 063716549.1 - Leptolyngbya valderiana
WP 012500045.1 - Chloroherpeton thalassium
WP 011422979.1 - Anaeromyxobacter dehalogenans
WP 041464105.1 - Pelodictyon luteolum
WP 012675888.1 - Persephonella marina
S2 bin6 00592 - Epsilonproteobacteria
WP 007474247.1 - Caminibacter mediatlanticus
WP 084275089.1 - Nitratiruptor tergarcus
S2 bin54 00486 - Bacteroidetes
WP 015677801.1 - Leptospira yanagawae
WP 013013834.1 - Meiothermus ruber
WP 007572036.1 - Patulibacter medicamentivorans
S2 bin54 01610 - Bacteroidetes
S2 bin54 02128 - Bacteroidetes
WP 112085470.1 - Flavobacterium lacus
WP 101308588.1 - Labilibaculum manganireducens
WP 038247978.1 - Ghiorsea bivora
WP 010851189.1 - Tetrasphaera elongata
S2 bin31 00198 - Epsilonproteobacteria
S2 bin6 00027 - Epsilonproteobacteria
WP 013075664.1 - Kyrpidia tusciae
WP 007546197.1 - Sulfurihydrogenibium yellowstonense
WP 041470237.1 - Chlorobium phaeovibrioides
WP 006928554.1 - Caldithrix abyssi
WP 036533293.1 - Neosynechococcus sphagnicola
WP 009545423.1 - Crocosphaera subtropica
WP 063714927.1 - Leptolyngbya valderiana
WP 011056143.1 - Thermosynechococcus elongatus
WP 012963971.1 - Hydrogenobacter thermophilus
WP 053381235.1 - Nitrospira moscoviensis
WP 028989416.1 - Thermithiobacillus tepidarius
WP 018077141.1 - Thiobacillus denitrificans
WP 023265754.1 - Shewanella decolorationis
WP 004286847.1 - Francisella philomiragia
S1 bin3 00913 - Gammaproteobacteria
S2 bin22 01281 - Gammaproteobacteria
WP 014108326.1 - Glaciecola nitratireducens
WP 012536761.1 - Acidithiobacillus ferrooxidans
WP 049724352.1 - Wenzhouxiangella marina
WP 018293624.1 - Mariprofundus ferrooxydans
S6 bin19 00990 - Gammaproteobacteria
WP 068168305.1 - Hydrogenophaga taeniospiralis
WP 011942208.1 - Acidiphilium cryptum
S2 bin25 01392 - Alphaproteobacteria
0.1

Supplementary Figure 4 Maximum-likelihood tree of amino acid sequences of the sulfide-quinone oxidoreductase (Sqr), a marker for sulfide oxidation. The tree shows sequences from cold seep metagenome-assembled genomes obtained in this study (blue) alongside representative reference sequences (black). The subgroup of each reference sequence is denoted. The tree was constructed using the JTT matrix-based model, used all sites, and was bootstrapped with 50 replicates and midpoint-rooted.

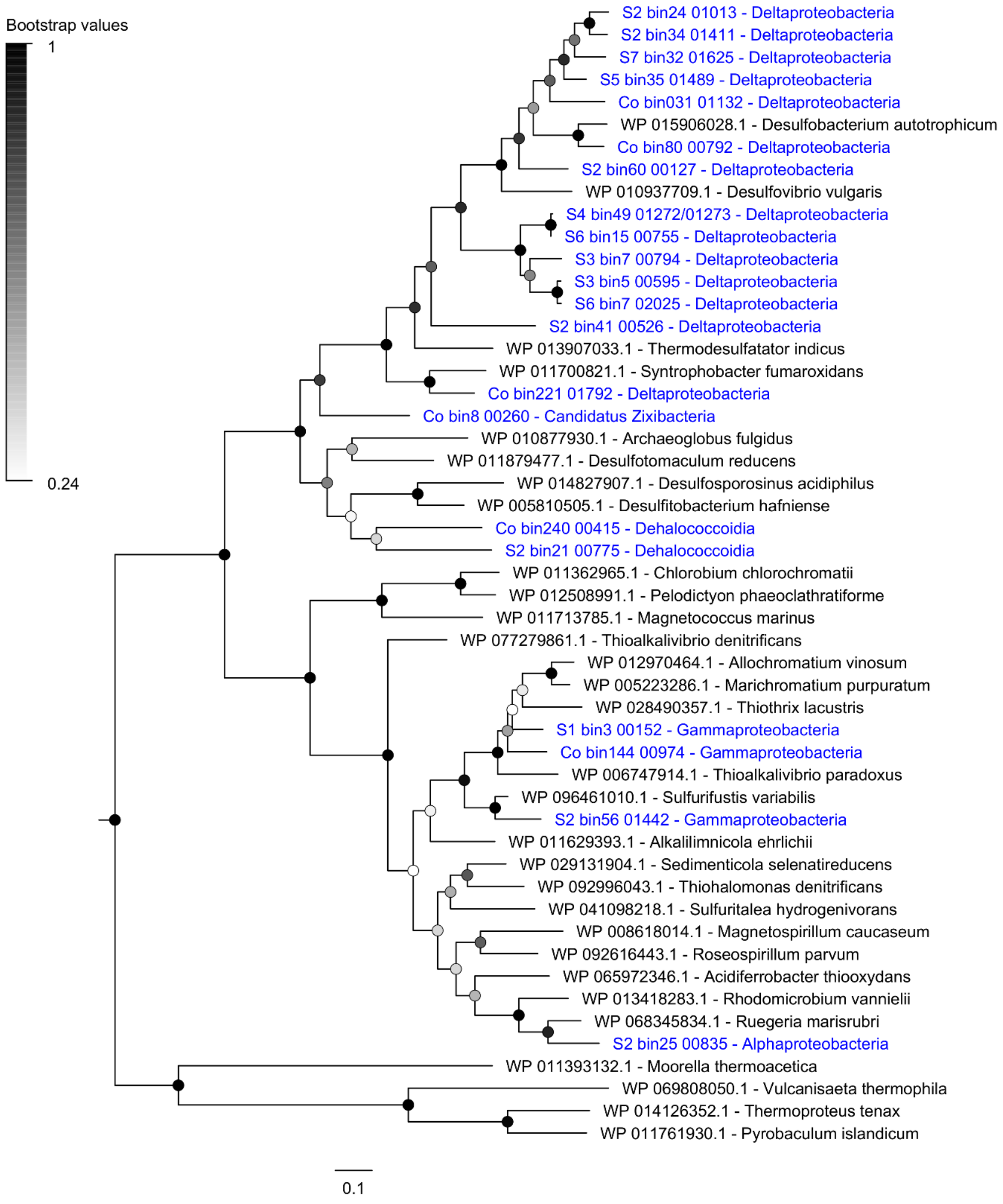

Supplementary Figure 5 Maximum-likelihood tree of amino acid sequences of dissimilatory sulfite reductase A subunit (DsrA). The tree shows sequences from cold seep metagenome-assembled genomes obtained in this study (blue) alongside representative reference sequences (black). This enzyme is a marker for dissimilatory sulfite reduction (top and bottom major clades; *Deltaproteobacteria*, Zixibacteria, *Dehalococcoidia* bins) and sulfide oxidation (middle clade; *Gammaproteobacteria* and Alphaproteobacteria bins). The tree was constructed using the JTT matrix-based model, used all sites, and was bootstrapped with 50 replicates and midpoint-rooted.

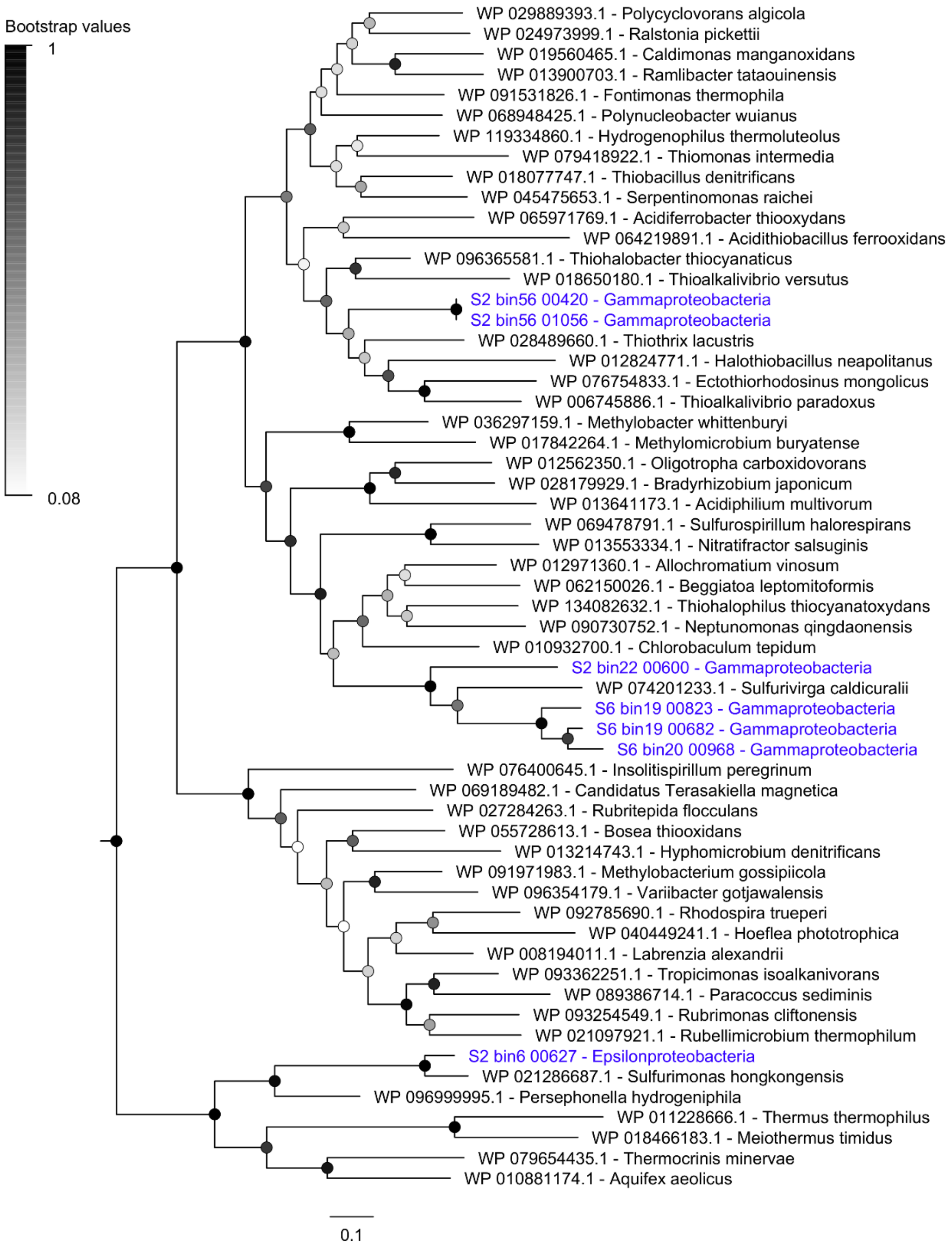

Supplementary Figure 6 Maximum-likelihood tree of amino acid sequences of the thiosulfohydrolase (SoxB), a marker for thiosulfate oxidation. The tree shows sequences from cold seep metagenome-assembled genomes obtained in this study (blue) alongside representative reference sequences (black). The subgroup of each reference sequence is denoted. The tree was constructed using the JTT matrix-based model, used all sites, and was bootstrapped with 50 replicates and midpoint-rooted.

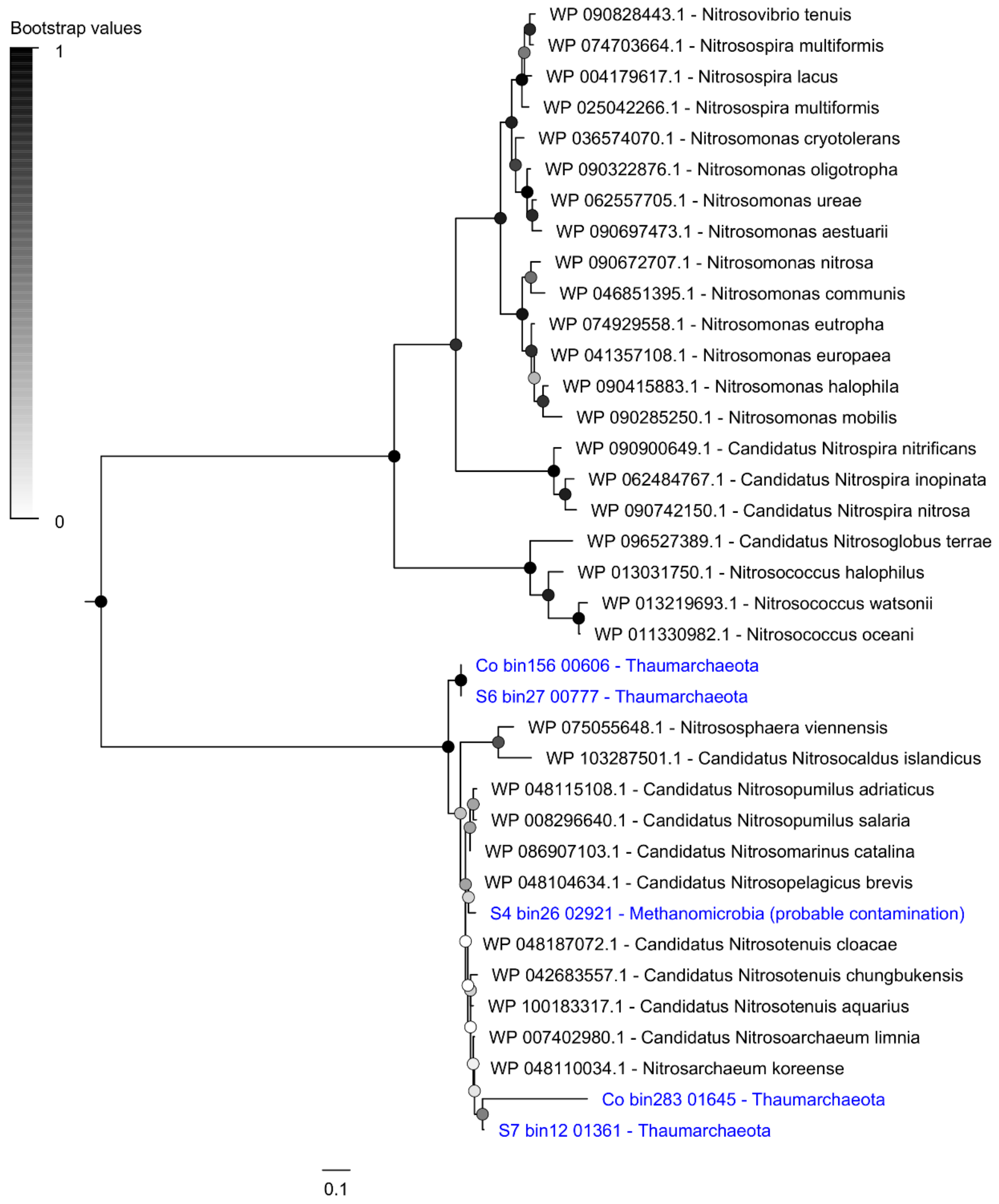

Supplementary Figure 7 Maximum-likelihood tree of amino acid sequences of ammonia monooxygenase A subunit (AmoA), a marker for ammonia oxidation during aerobic nitrification. The tree shows sequences from cold seep metagenome-assembled genomes obtained in this study (blue) alongside representative reference sequences (black). The tree was constructed using the JTT matrix-based model, used all sites, and was bootstrapped with 50 replicates and midpoint-rooted.

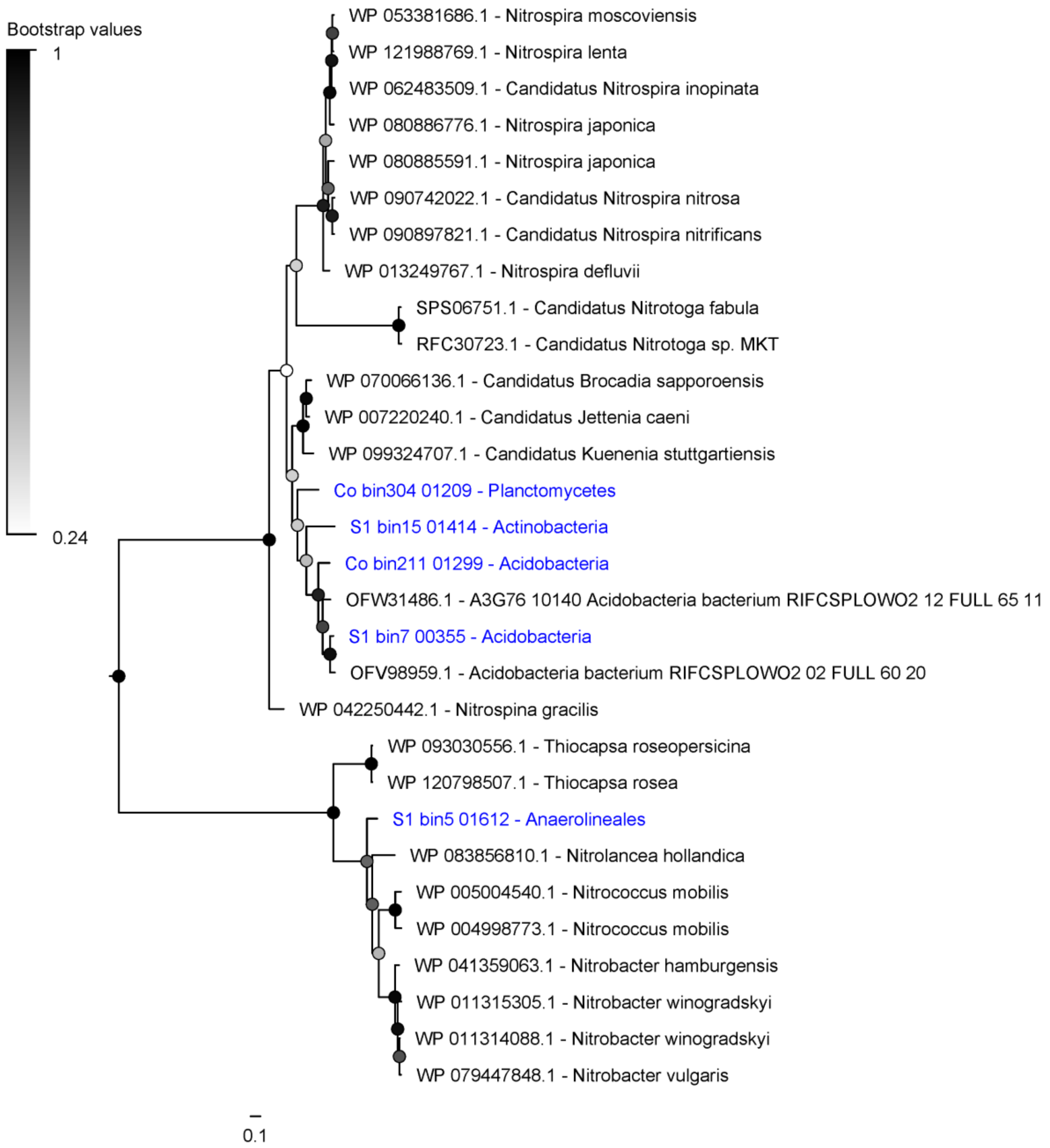

Supplementary Figure 8 Maximum-likelihood tree of amino acid sequences of the nitrite oxidoreductase A subunit (NxrA), a marker for nitrite oxidation during aerobic nitrification. The tree shows sequences from cold seep metagenome-assembled genomes obtained in this study (blue) alongside representative reference sequences (black). The subgroup of each reference sequence is denoted. The tree was constructed using the JTT matrix-based model, used all sites, and was bootstrapped with 50 replicates and midpoint-rooted.

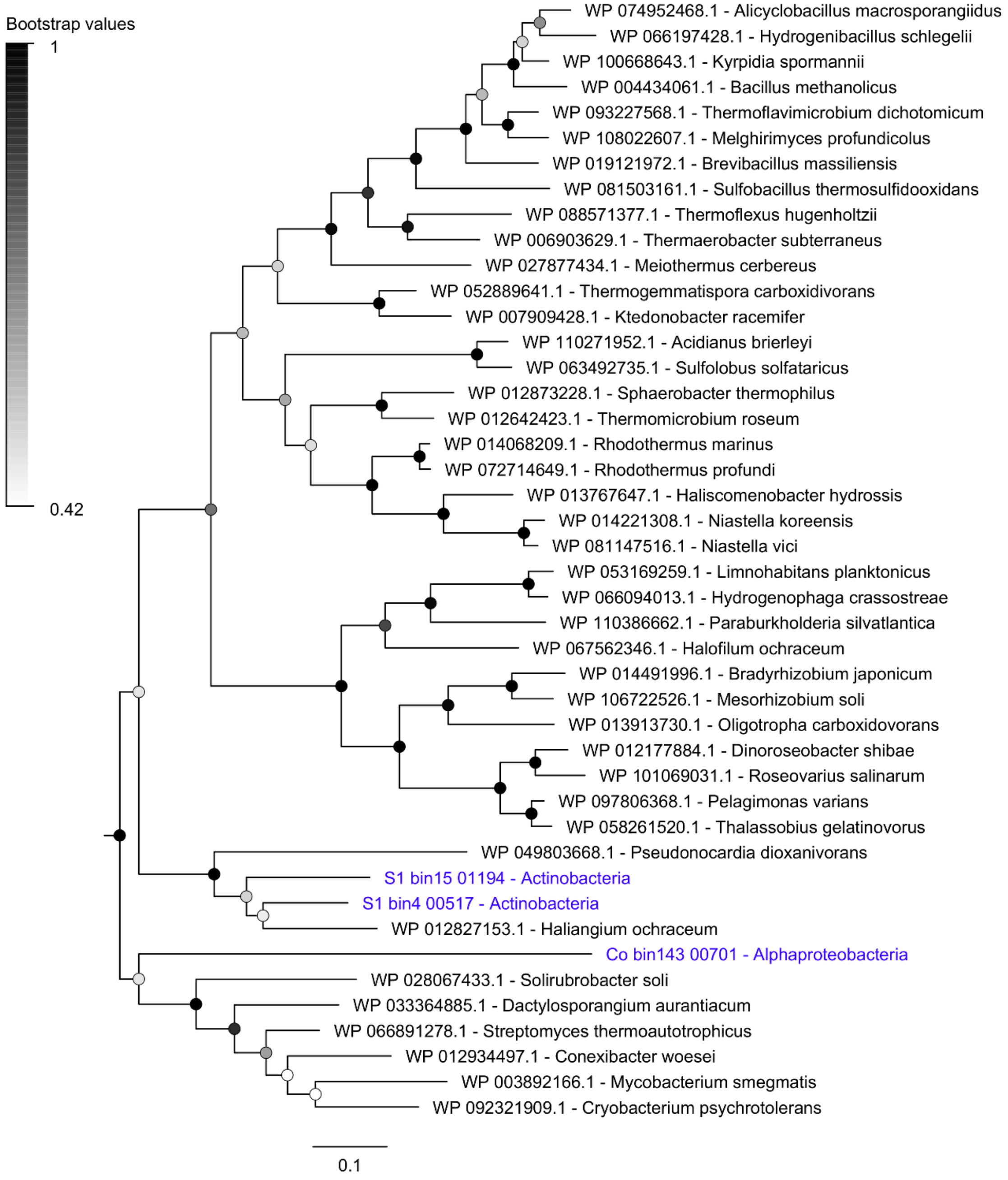

Supplementary Figure 9 Maximum-likelihood tree of amino acid sequences of carbon monoxide dehydrogenase large subunit (CoxL), a marker for aerobic carbon monoxide oxidation. The tree shows sequences from cold seep metagenome-assembled genomes obtained in this study (blue) alongside representative reference sequences (black). The tree was constructed using the JTT matrix-based model, used all sites, and was bootstrapped with 50 replicates and midpoint-rooted.

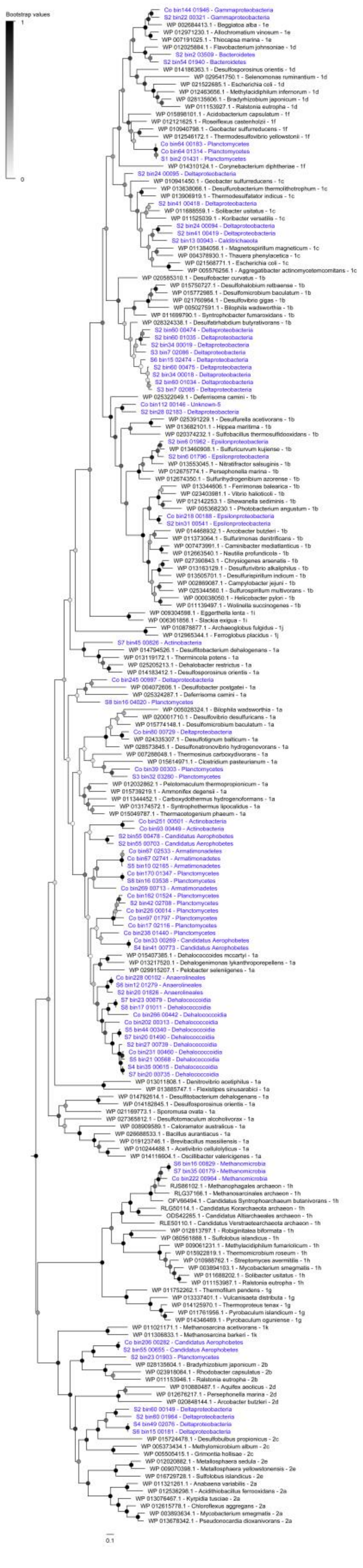

Supplementary Figure 10 Maximum-likelihood tree of amino acid sequences of group 1 and 2 [NiFe]-hydrogenase large subunits, a marker for hydrogen oxidation during respiratory processes. The tree shows sequences from cold seep metagenome-assembled genomes obtained in this study (blue) alongside representative reference sequences (black). The subgroup of each reference sequence is denoted according to the HydDB classification scheme. The tree was constructed using the JTT matrix-based model, used all sites, and was bootstrapped with 50 replicates and midpoint-rooted.

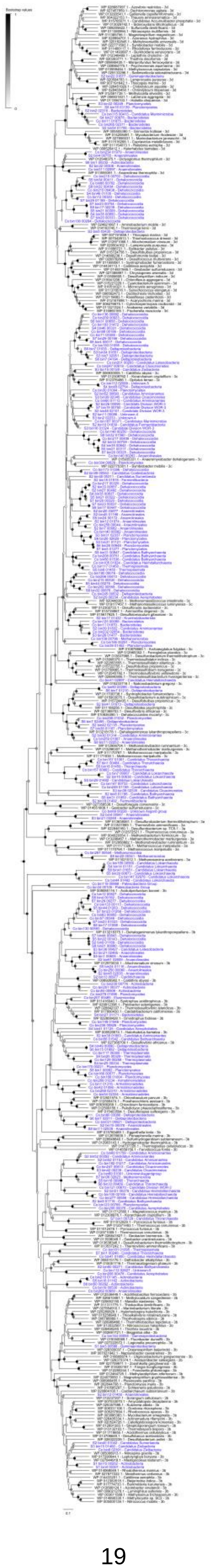

Supplementary Figure 11 Neighbour-joining tree of amino acid sequences of the group 3 [NiFe]-hydrogenase large subunit, a marker for bidirectional hydrogen metabolism during various processes. The tree shows sequences from cold seep metagenome-assembled genomes obtained in this study (blue) alongside representative reference sequences (black). The subgroup of each reference sequence is denoted according to the HydDB classification scheme. The tree was constructed using the Poisson model with gaps treated with pairwise deletion, was bootstrapped with 50 replicates, and was midpoint-rooted. To enable neighbour-joining, 54 incomplete sequences of the original 298 hits were excluded from this analysis.

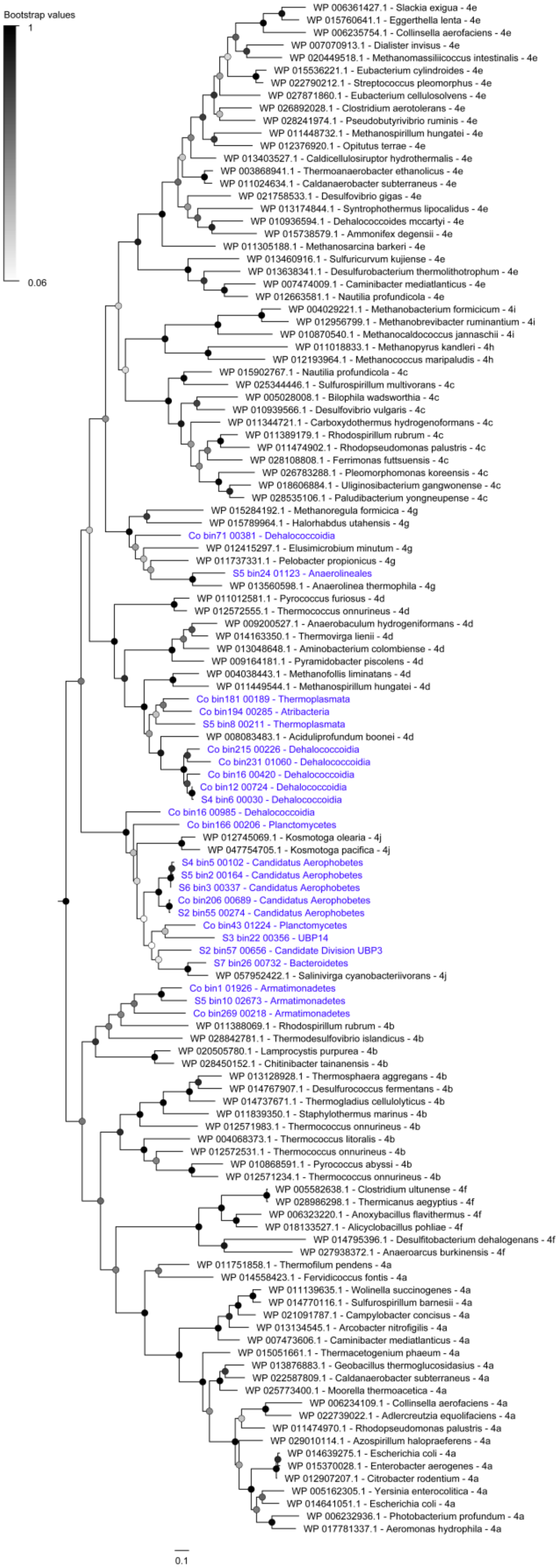
Bootstrap values
1
0.06
WP 006361427.1 - Slackia exigua - 4e
WP 015760641.1 - Eggerthella lenta - 4e
WP 006235754.1 - Collinsella aerofaciens - 4e
WP 007070913.1 - Dialister invisus - 4e
WP 020449518.1 - Methanomassiliicoccus intestinalis - 4e
WP 015536221.1 - Eubacterium cylindroides - 4e
WP 022790212.1 - Streptococcus pleomorphus - 4e
WP 027871860.1 - Eubacterium cellulosolvens - 4e
WP 026892028.1 - Clostridium aerotolerans - 4e
WP 028241974.1 - Pseudobutyrivibrio ruminis - 4e
WP 011448732.1 - Methanospirillum hungatei - 4e
WP 012376920.1 - Opitutus terrae - 4e
WP 013403527.1 - Caldicellulosiruptor hydrothermalis - 4e
WP 003868941.1 - Thermoanaerobacter ethanolicus - 4e
WP 011024634.1 - Caldanaerobacter subterraneus - 4e
WP 021758533.1 - Desulfovibrio gigas - 4e
WP 013174844.1 - Syntrophothermus lipocalidus - 4e
WP 010936594.1 - Dehalococcoides mccartyi - 4e
WP 015738579.1 - Ammonifex degensii - 4e
WP 011305188.1 - Methanosarcina barkeri - 4e
WP 013460916.1 - Sulfuricurvum kujiense - 4e
WP 013638341.1 - Desulfurobacterium thermolithotrophum - 4e
WP 007474009.1 - Caminibacter mediatlanticus - 4e
WP 012663581.1 - Nautilia profundicola - 4e
WP 004029221.1 - Methanobacterium formicicum - 4i
WP 012956799.1 - Methanobrevibacter ruminantium - 4i
WP 010870540.1 - Methanocaldococcus jannaschii - 4i
WP 011018833.1 - Methanopyrus kandleri - 4h
WP 012193964.1 - Methanococcus maripaludis - 4h
WP 015902767.1 - Nautilia profundicola - 4c
WP 025344446.1 - Sulfurospirillum multivorans - 4c
WP 005028008.1 - Bilophila wadsworthia - 4c
WP 010939566.1 - Desulfovibrio vulgaris - 4c
WP 011344721.1 - Carboxydothermus hydrogenoformans - 4c
WP 011389179.1 - Rhodospirillum rubrum - 4c
WP 011474902.1 - Rhodopseudomonas palustris - 4c
WP 028108808.1 - Ferrimonas futtsuensis - 4c
WP 026783288.1 - Pleomorphomonas koreensis - 4c
WP 018606884.1 - Uliginosibacterium gangwonense - 4c
WP 028535106.1 - Paludibacterium yongneupense - 4c
WP 015284192.1 - Methanoregula formicica - 4g
WP 015789964.1 - Halorhabdus utahensis - 4g
Co bin71 00381 - Dehalococcoidia
WP 012415297.1 - Elusimicrobium minutum - 4g
WP 011737331.1 - Pelobacter propionicus - 4g
S5 bin24 01123 - Anaerolineales
WP 013560598.1 - Anaerolinea thermophila - 4g
WP 011012581.1 - Pyrococcus furiosus - 4d
WP 012572555.1 - Thermococcus onnurineus - 4d
WP 009200527.1 - Anaerobaculum hydrogeniformans - 4d
WP 014163350.1 - Thermovirga lienii - 4d
WP 013048648.1 - Aminobacterium colombiense - 4d
WP 009164181.1 - Pyramidobacter piscolens - 4d
WP 004038443.1 - Methanofollis liminatans - 4d
WP 011449544.1 - Methanospirillum hungatei - 4d
Co bin181 00189 - Thermoplasmata
Co bin194 00285 - Atribacteria
S5 bin8 00211 - Thermoplasmata
WP 008083483.1 - Aciduliprofundum boonei - 4d
Co bin215 00226 - Dehalococcoidia
Co bin231 01060 - Dehalococcoidia
Co bin16 00420 - Dehalococcoidia
Co bin12 00724 - Dehalococcoidia
S4 bin6 00030 - Dehalococcoidia
Co bin16 00985 - Dehalococcoidia
Co bin166 00206 - Planctomycetes
WP 012745069.1 - Kosmotoga olearia - 4j
WP 047754705.1 - Kosmotoga pacifica - 4j
S4 bin5 00102 - Candidatus Aerophobetes
S5 bin2 00164 - Candidatus Aerophobetes
S6 bin3 00337 - Candidatus Aerophobetes
Co bin206 00689 - Candidatus Aerophobetes
S2 bin55 00274 - Candidatus Aerophobetes
Co bin43 01224 - Planctomycetes
S3 bin22 00356 - UBP14
S2 bin57 00656 - Candidate Division UBP3
S7 bin26 00732 - Bacteroidetes
WP 057952422.1 - Salinivirga cyanobacteriivorans - 4j
Co bin1 01926 - Armatimonadetes
S5 bin10 02673 - Armatimonadetes
Co bin269 00218 - Armatimonadetes
WP 011388069.1 - Rhodospirillum rubrum - 4b
WP 028842781.1 - Thermodesulfovibrio islandicus - 4b
WP 020505780.1 - Lamprocystis purpurea - 4b
WP 028450152.1 - Chitinibacter tainanensis - 4b
WP 013128928.1 - Thermosphaera aggregans - 4b
WP 014767907.1 - Desulfurococcus fermentans - 4b
WP 014737671.1 - Thermogladius cellulolyticus - 4b
WP 011839350.1 - Staphylothermus marinus - 4b
WP 012571983.1 - Thermococcus onnurineus - 4b
WP 004068373.1 - Thermococcus litoralis - 4b
WP 012572531.1 - Thermococcus onnurineus - 4b
WP 010868591.1 - Pyrococcus abyssi - 4b
WP 012571234.1 - Thermococcus onnurineus - 4b
WP 005582638.1 - Clostridium ultunense - 4f
WP 028986298.1 - Thermicanus aegyptius - 4f
WP 006323220.1 - Anoxybacillus flavithermus - 4f
WP 018133527.1 - Alicyclobacillus pohliae - 4f
WP 014795396.1 - Desulfitobacterium dehalogenans - 4f
WP 027938372.1 - Anaeroarcus burkinensis - 4f
WP 011751858.1 - Thermofilum pendens - 4a
WP 014558423.1 - Fervidicoccus fontis - 4a
WP 011139635.1 - Wolinella succinogenes - 4a
WP 014770116.1 - Sulfurospirillum barnesii - 4a
WP 021091787.1 - Campylobacter concisus - 4a
WP 013134545.1 - Arcobacter nitrofigilis - 4a
WP 007473606.1 - Caminibacter mediatlanticus - 4a
WP 015051661.1 - Thermacetogenium phaeum - 4a
WP 013876883.1 - Geobacillus thermoglucosidasius - 4a
WP 022587809.1 - Caldanaerobacter subterraneus - 4a
WP 025773400.1 - Moorella thermoacetica - 4a
WP 006234109.1 - Collinsella aerofaciens - 4a
WP 022739022.1 - Adlercreutzia equolifaciens - 4a
WP 011474970.1 - Rhodopseudomonas palustris - 4a
WP 029010114.1 - Azospirillum halopraeferens - 4a
WP 014639275.1 - Escherichia coli - 4a
WP 015370028.1 - Enterobacter aerogenes - 4a
WP 012907207.1 - Citrobacter rodentium - 4a
WP 005162305.1 - Yersinia enterocolitica - 4a
WP 014641051.1 - Escherichia coli - 4a
WP 006232936.1 - Photobacterium profundum - 4a
WP 017781337.1 - Aeromonas hydrophila - 4a
0.1

Supplementary Figure 12 Maximum-likelihood tree of amino acid sequences of the group 4 [NiFe]-hydrogenase large subunit, a marker primarily for hydrogen production during fermentative and respiratory processes. The tree shows sequences from cold seep metagenome-assembled genomes obtained in this study (blue) alongside representative reference sequences (black). The subgroup of each reference sequence is denoted according to the HydDB classification scheme. The tree was constructed using the JTT matrix-based model, used all sites, and was bootstrapped with 50 replicates and midpoint-rooted.

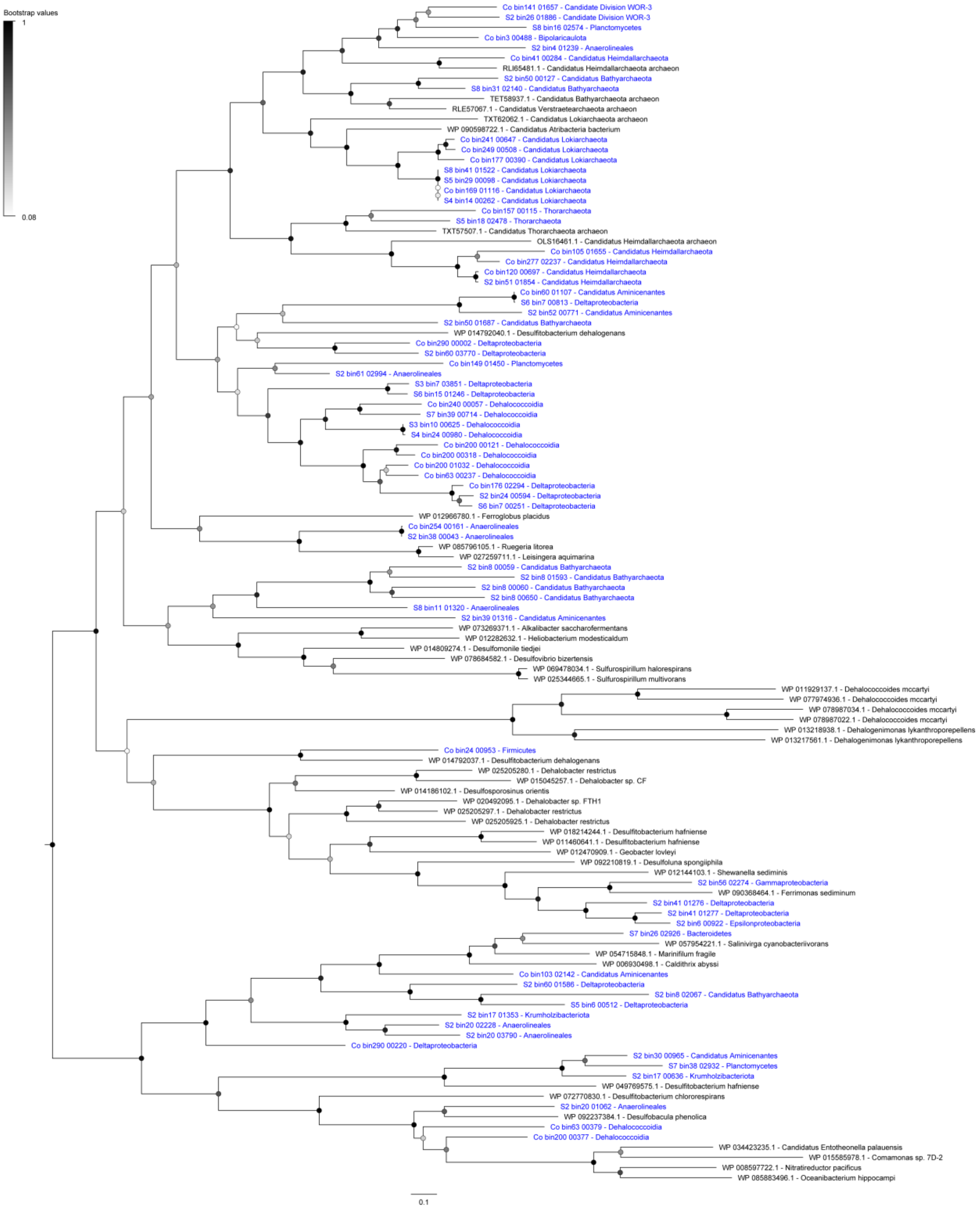

Supplementary Figure 13 Maximum-likelihood tree of amino acid sequences of the reductive dehalogenase A subunit (RdhA), a marker for reductive dehalogenation. The tree shows sequences from cold seep metagenome-assembled genomes obtained in this

study (blue) alongside representative reference sequences (black). The subgroup of each reference sequence is denoted. The tree was constructed using the JTT matrix-based model, used all sites, and was bootstrapped with 50 replicates and midpoint-rooted.

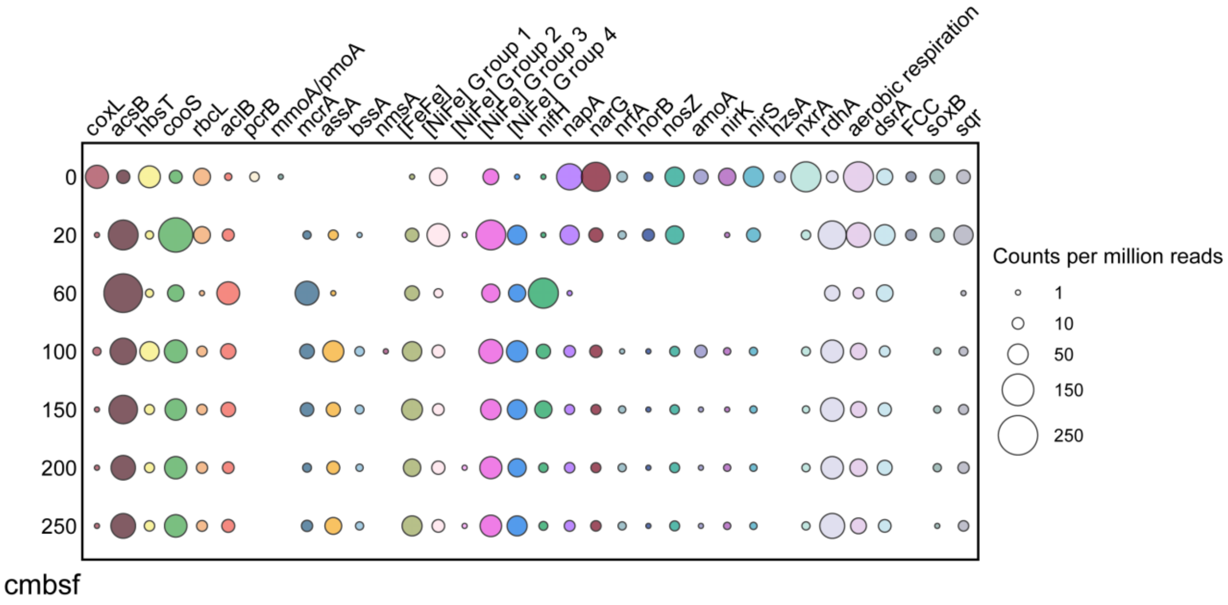

Supplementary Figure 14 Depth-differentiated metabolic capacity based on normalized short-read counts of genes encoding the catalytic subunits of key metabolic enzymes in unassembled quality-filtered reads. Genes are marked by different colors. Details on the annotation of the enzymes are presented in **Supplementary Data 5**.

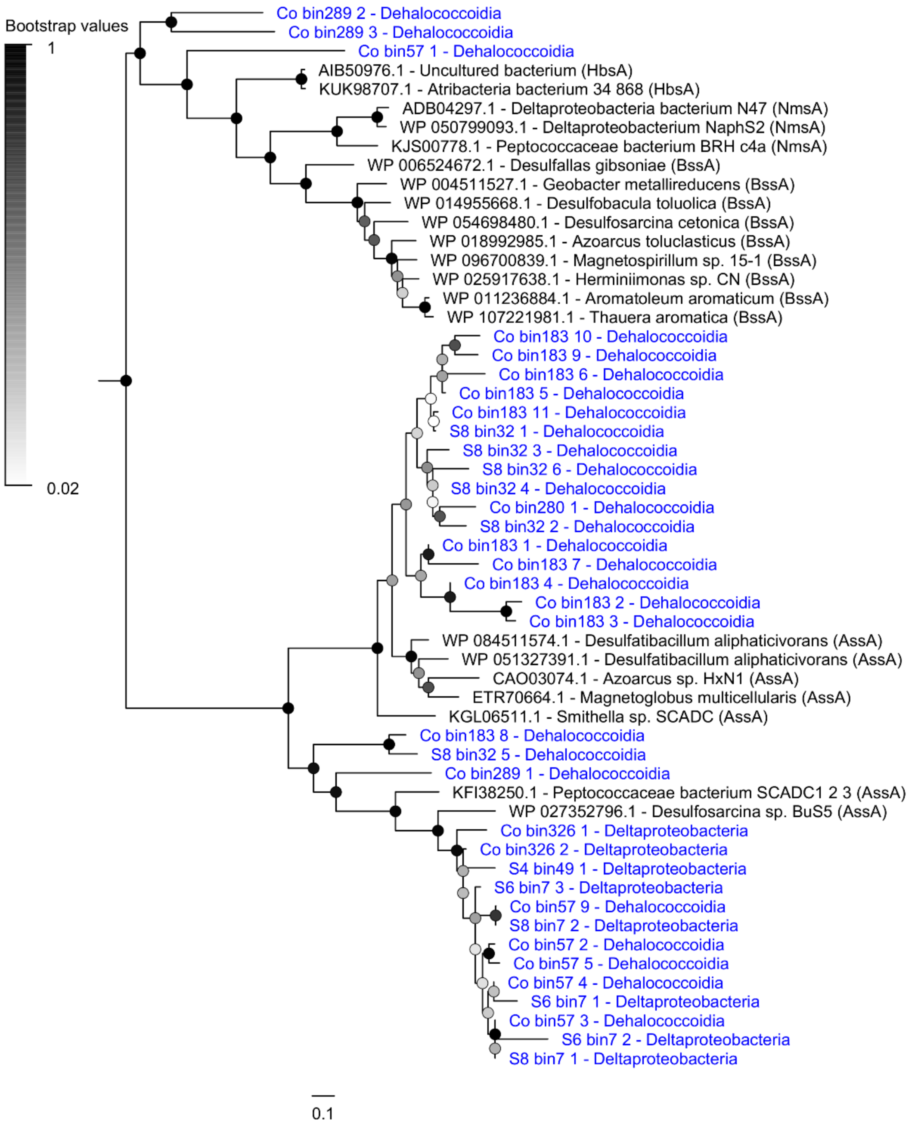

Supplementary Figure 15 Maximum-likelihood tree of amino acid sequences of catalytic subunits of canonical fumarate-adding enzymes, which are markers for anaerobic hydrocarbon degradation. The tree shows sequences from cold seep metagenome-assembled genomes obtained in this study (blue) alongside representative reference sequences (black). Different clades correspond to alkylsuccinate synthases (AssA), benzylsuccinate synthases (BssA), naphthylmethylsuccinate synthases (NmsA), and hydroxybenzylsuccinate synthases (HbsA). The tree was constructed using the JTT matrix-based model, used all sites, and was bootstrapped with 50 replicates and midpoint-rooted.

Supplementary references

1. Lombard, V., Golaconda Ramulu, H., Drula, E., Coutinho, P.M. & Henrissat, B. The carbohydrate-active enzymes database (CAZy) in 2013. *Nucleic Acids Res* **42**, D490-495 (2014).
2. Greening, C. et al. Genomic and metagenomic surveys of hydrogenase distribution indicate H_2 is a widely utilised energy source for microbial growth and survival. *ISME J* **10**, 761 (2015).
3. Müller, A.L., Kjeldsen, K.U., Rattei, T., Pester, M. & Loy, A. Phylogenetic and environmental diversity of DsrAB-type dissimilatory (bi)sulfite reductases. *ISME J* **9**, 1152-1165 (2015).
4. Kublik, A. et al. Identification of a multi-protein reductive dehalogenase complex in *Dehalococcoides mccartyi* strain CBDB1 suggests a protein-dependent respiratory electron transport chain obviating quinone involvement. *Environ Microbiol* **18**, 3044-3056 (2016).
5. Søndergaard, D., Pedersen, C.N. & Greening, C. HydDB: A web tool for hydrogenase classification and analysis. *Sci Rep* **6**, 34212 (2016).
