# Supplementary material for: Thermogenic hydrocarbon biodegradation by diverse depth-stratified microbial populations at a Scotian Basin cold seep
Source: Nat Commun. 2020 Nov 17;11:5825. doi: 10.1038/s41467-020-19648-2 (PMC7673041; doi:10.1038/s41467-020-19648-2)
Supplement: Supplementary file 2 — Description of Additional Supplementary Files [file 41467_2020_19648_MOESM2_ESM.pdf]

Description of Additional Supplementary Files

File Name: Supplementary Data 1
Description: Archaeal (a) and bacterial (b) OTU tables based on 16S rRNA gene amplicon sequencing.

File Name: Supplementary Data 2
Description: Genome information for 376 MAGs, including (1) genome completeness and contamination estimates (determined by CheckM), (2) taxonomic assignment of MAGs based on 16S rRNA gene sequences where possible, (3) taxonomic assignment of MAGs based on GTDB-tK classification, and (4) taxonomic assignment of MAGs based on NCBI taxonomy determined by a phylogenetic analysis of 43 marker genes.

File Name: Supplementary Data 3
Description: Statistics of metagenome assemblies. Data were generated using QUAST (default parameters). All statistics are based on contigs of size >= 500 bp, unless otherwise noted (e.g., "# contigs (>= 0 bp)" and "Total length (>= 0 bp)" include all contigs).

File Name: Supplementary Data 4

Description: Newick tree file for the maximum likelihood phylogenetic tree of 376 MAGs based on 43 concatenated protein-coding genes in Supplementary Figure 2. Reference genomes for relatives were accessed from NCBI GenBank. The tree was built using RAxML with the PROTGAMMALG model.

File Name: Supplementary Data 5
Description: Summary for occurrence of various functions of interest encoded within each MAG. Pathways are scaled from 0 to 1, where 1 represents that the genome contains all genes for the function of interest. Detailed gene list for each pathway can be found at: https://github.com/bjtully/BioData/blob/master/KEGGDecoder.

File Name: Supplementary Data 6

Description: Summary of carbohydrate-active enzymes (CAZymes) detected in 376 MAGs. Total numbers for carbohydrate esterases (CE), glycoside hydrolases (GH) and polysaccharide lyases (PL), as well as corresponding raw outputs from the dbCAN2 webserver, are shown.

File Name: Supplementary Data 7

Description: Total number of peptidases detected in each MAG. Total number of peptidases identified using the Pfam HMM models related to the MEROPS peptidase database.

File Name: Supplementary Data 8

Description: Details for total number of genes related to the beta oxidation pathway in each genome using the KAAS webserver.

File Name: Supplementary Data 9

Description: Details for identification of genes encoding for (1) different types of hydrogenases and (2) reductive dehalogenases in each MAG. Hydrogenases were classified using the hydrogenase classifier HydDB, and *rdhA* genes were detected using TIGR02486.

File Name: Supplementary Data 10

Description: Details for identification of genes in genomes containing *mcrA* genes based on presence/absence matrix. Also include detailed genome statistics on these genomes.

File Name: Supplementary Data 11

Description: Details for identification of genes in genomes containing *assA* genes based on presence/absence matrix. Also include detailed genome statistics on these genomes.

File Name: Supplementary Data 12

Description: Details for identification of genes in genomes containing *bcr* genes based on presence/absence matrix. Also include detailed genome statistics on these genomes. Only MAGs contain at least two further genes in this pathway are discussed in the main text.

File Name: Supplementary Data 13

Description: Relative abundances and replication rates of microorganisms based on representative MAGs for each species group. The relative abundances (%) were determined by mapping each MAG against quality-filtered metagenome reads using CoverM. *In situ* replication rate measurements were determined by iRep.

File Name: Supplementary Data 14

Description: Details for identification of multiheme c-type cytochromes in archaeal MAGs harboring *mcrA* genes.

File Name: Supplementary Data 15

Description: Sequences for amino acids that used to construct *mcrA* trees in Figure 4b.
