# Supplementary material for: Thermogenic hydrocarbon biodegradation by diverse depth-stratified microbial populations at a Scotian Basin cold seep
Source: Nat Commun. 2020 Nov 17;11:5825. doi: 10.1038/s41467-020-19648-2 (PMC7673041; doi:10.1038/s41467-020-19648-2)
Supplement: Supplementary file 18 — Reporting Summary [file 41467_2020_19648_MOESM18_ESM.pdf]

nature research

Corresponding author(s): Xiyang Dong and Casey Hubert

Last updated by author(s): Sep 22, 2020

Reporting Summary

Nature Research wishes to improve the reproducibility of the work that we publish. This form provides structure for consistency and transparency in reporting. For further information on Nature Research policies, see Authors & Referees and the Editorial Policy Checklist .

Statistics

For all statistical analyses, confirm that the following items are present in the figure legend, table legend, main text, or Methods section.

n/a	Confirmed	
☐	☒	The exact sample size (n) for each experimental group/condition, given as a discrete number and unit of measurement
☐	☒	A statement on whether measurements were taken from distinct samples or whether the same sample was measured repeatedly
☒	☐	The statistical test(s) used AND whether they are one- or two-sided<br>*Only common tests should be described solely by name; describe more complex techniques in the Methods section.*
☒	☐	A description of all covariates tested
☒	☐	A description of any assumptions or corrections, such as tests of normality and adjustment for multiple comparisons
☒	☐	A full description of the statistical parameters including central tendency (e.g. means) or other basic estimates (e.g. regression coefficient) AND variation (e.g. standard deviation) or associated estimates of uncertainty (e.g. confidence intervals)
☒	☐	For null hypothesis testing, the test statistic (e.g. F, t, r) with confidence intervals, effect sizes, degrees of freedom and P value noted<br>*Give P values as exact values whenever suitable.*
☒	☐	For Bayesian analysis, information on the choice of priors and Markov chain Monte Carlo settings
☒	☐	For hierarchical and complex designs, identification of the appropriate level for tests and full reporting of outcomes
☒	☐	Estimates of effect sizes (e.g. Cohen's d, Pearson's r), indicating how they were calculated

Our web collection on statistics for biologists contains articles on many of the points above.

Software and code

Policy information about availability of computer code

Data collection	Agilent 7890A RGA Gas Chromatograph, Trace GC2000, Thermo Finnigan Delta plus XP isotope ratio mass spectrometer, Agilent 7890A RGA Gas Chromatograph, Micromass ProSpec-Q instrument, Eurovector EA3028 connected to a Nu Horizon IRMS, Dionex ICS-5000 reagent-free ion chromatography system, Thermo RS3000 HPLC fitted with an Ultimate 3000 UV detector, MAVEN software v3.6.0, Ultra High-Performance Liquid Chromatography (UHPLC) using a hydrophilic interaction liquid chromatography column (Syncronis HILIC, Thermo Fisher), PowerSoil DNA Isolation Kit (12888-50, QIAGEN), PowerSoil DNA Isolation Kit (12888-50, QIAGEN), MiSeq benchtop sequencer, NextSeq 500 System, Thermo Scientific PikoReal Real-Time PCR Instrument.
Data analysis	MetaAmp v2.0, PikoReal software v2.2, SingleM v0.12.1, MEGAHIT v1.1.3, metaSPAdes v3.11.0, BBTools (https://sourceforge.net/projects/bbmap/), CheckM v1.0.8, Prodigal v2.6.3, metaWRAP v1.0, maxbin2, metabat1, metabat2, dRep v2.2.1, GhostKOALA tool v2.2, BamM v1.7.3, CoverM v0.4.0, Bowtie2, iRep v1.10, DIAMOND v0.9.26, MetaErg v1.0.0 (https://github.com/xiaoli-dong/metaerg), BLASTp, KEGG-Decoder and KEGG-Expander v1.0 (https://github.com/bjtully/BioData/tree/master/KEGGDecoder), RAxML v8, IQ-Tree v1.6.12, TrimAL v1.2, MUSCLE v3.5, MEGA7, hydrogenase classifier HydDB (https://services.birc.au.dk/hyddb/), Batch CD-Search (https://www.ncbi.nlm.nih.gov/Structure/bwrpsb/bwrpsb.cgi), phyloFlash v3.1 (https://hrgv.github.io/phyloFlash/), dbCAN2 web server (http://bcb.unl.edu/dbCAN2/), anvi'o v5, SILVA ACT (https://www.arb-silva.de/aligner/), GTDB-Tk v0.3.3 (using GTDB R04-RS89), Psortb v3.0, ClustalW v1.2.2.

For manuscripts utilizing custom algorithms or software that are central to the research but not yet described in published literature, software must be made available to editors/reviewers. We strongly encourage code deposition in a community repository (e.g. GitHub). See the Nature Research guidelines for submitting code & software for further information.

Data

Policy information about availability of data

All manuscripts must include a data availability statement. This statement should provide the following information, where applicable:

- Accession codes, unique identifiers, or web links for publicly available datasets
- A list of figures that have associated raw data
- A description of any restrictions on data availability

The links to the databases used in this study are listed below: Silva database (release 132): https://www.arb-silva.de/documentation/release-132/; NCBI Taxonomy database: https://www.ncbi.nlm.nih.gov/taxonomy; Pfam: https://pfam.xfam.org/; TIGRfam: https://tigrfams.jcvi.org/cgi-bin/index.cgi; KEGG GENES Database: https://www.genome.jp/kegg/genes.html; the custom database for anaerobic hydrocarbon degradation: https://www.nature.com/articles/s41467-019-09747-0#additional-information. DNA sequences have been deposited in NCBI BioProject databases with accession number PRJNA598277 (https://www.ncbi.nlm.nih.gov/bioproject/?term=PRJNA598277). Individual assembly for metagenome-assembled genomes can be found at figshare (https://figshare.com/s/bee9fd40f45054e71e8b). The authors declare that all other data supporting the findings of this study are available within the article and its supplementary information files, or from the corresponding authors upon request.

Field-specific reporting

Please select the one below that is the best fit for your research. If you are not sure, read the appropriate sections before making your selection.

☐ Life sciences ☐ Behavioural & social sciences ☒ Ecological, evolutionary & environmental sciences

For a reference copy of the document with all sections, see nature.com/documents/nr-reporting-summary-flat.pdf

Ecological, evolutionary & environmental sciences study design

All studies must disclose on these points even when the disclosure is negative.

Study description	This study provides a detailed analysis of a 3.44-meter-long piston core taken from the seabed of the Scotian slope (43.010478 N, 60.211777 W) in 2306 m water depth. We combine geophysical, geochemical and metabolomic analyses with gene- and genome-centric metagenomics to understand the communities and processes responsible for anaerobic oxidation of different hydrocarbons, as well as other associated metabolisms, at this newly discovered deep sea cold seep.
Research sample	The coring location was chosen based on seismic interpretation using Petrel, that focused on identifying amplitude anomalies for direct hydrocarbon indicators. One such indicator at this location was inferred to be associated with a possible seabed seep by interpreting a subsurface hydrocarbon migration pathway in the form of a fault to surface. Being representative for cold seep sediments, this core was much lighter in colour than typical sediments recovered from that area. The core also contained firm to semi-firm concretions that possibly represent authigenic carbonates. In the bottom of the core, at 332-344 cm below the seafloor (cmbsf), gas hydrates were observed in frozen crystalized form and numerous gas bubbles escaped during retrieval. A strong sulfide odor was also detected during core retrieval and processing.
Sampling strategy	For subsampling from the core at each depth, no specific sample size calculation was performed. The sample size for each analysis was chosen based on the suggested weight or volume in the protocols (for geochemical characterization, and porewater sulfate and metabolite extraction) and DNA Isolation Kits (for DNA extraction). If their sizes were not sufficient, the donwstream analyses would not be able to produce any results. Details for geochemical characterization, porewater sulfate and metabolite extraction, and DNA extraction can be found in the manuscript.
Data collection	The core was taken using the piston coring system, the AGC Long Corer, which uses coupled core barrel sections in 10 ft (305 cm) lengths. Following piston coring, sediments subsamples were collected immediately from the base of the core and stored in gas-tight isojars flushed with gaseous nitrogen for headspace gas analysis. Multiple depths ranging from the deepest portion to within approximately a metre of the top of the core, were subsampled for geochemical analysis. Additional intervals were preserved separately for microbiological analyses. Detailed subsampling depths can be found in Tables 1 and 2 as well as Supplementary Table 1. X.D. processed metagenome data. J.E.R., S.M., R.A.G. and I.A.L. performed metabolomics analyses and data interpretation. D.C.C. was the chief scientist aboard the CCGS Hudson and was responsible for sediment sampling. M.F., J.W. and A.M. designed and performed petroleum geochemical analyses. N.M.M., D.C.C. and A.M. interpreted geophysical data. C.G. performed phylogenetic analysis of key metabolic genes. C.L. conducted amplicon sequencing and analyses. A. C. performed microbial diversity analyses. O.A. performed porewater sulfate measurements. S.W. and D.M. conducted qPCR analyses.
Timing and spatial scale	During Hudson 2016-011 Phase 2 for a piston coring cruise, on Sunday July 3, 2016, at a water depth of 2306 m, a 3.44-meter-long piston core was retrieved using the CCGS Hudson. This timing was chosen, as it was a good weather condition for sailing. This location was selected for sampling based on it was located near the crest of buried salt structures where faults are interpreted to approach the seabed; these faults may be conduits for hydrocarbon migration from depth. Site surveys were conducted in these areas using high resolution seismic reflection systems in order to pick specific targets for coring. The core length was related to the piston coring system and the physical condition of the site as described above.
Data exclusions	No data were excluded from the manuscript.
Reproducibility	For all the measured parameters by the instruments, they were all performed in at least for duplicates. All the attempts for the replications were successful. For all other data produced by the software, they were obtained automatically.

Randomization	For samples for each depth, they were homogenized and sub-sampled for the various treatments.
Blinding	Sampling and analysis were not performed in a blinded fashion. Given the nature of the analyses, this is unlikely to significantly bias the findings.

Did the study involve field work? ☒ Yes ☐ No

Field work, collection and transport

Field conditions	Samples were taken in the NW Atlantic deep sea (2.3 km water depth) using the CCGS Hudson. As we took samples from the deep sea subseafloor, the atmosphere temperature and other environmental conditions could not affect them. They were taken on Sunday July 3, 2016. This timing was chosen, as it was a good weather condition (temp min. 13 - max. 22, passing clouds) for sailing. This location was selected for sampling based on it was located near the crest of buried salt structures where faults are interpreted to approach the seabed; these faults may be conduits for hydrocarbon migration from depth. Site surveys were conducted in these areas using high resolution seismic reflection systems in order to pick specific targets for coring.
Location	This study provides a detailed analysis of a 3.44-meter-long piston core taken from the seabed of the Scotian slope (43.010478 N, 60.211777 W) in 2306 m water depth.
Access and import/export	Access to the field location was arranged prior to sampling. The selection of this area was based on satellite and seismic reflection data, which show that this area has strong evidence for seepage of thermogenic hydrocarbons with occurrences of high-pressure diapirs, polygonal faults, pockmarks, and gas chimneys. This piston coring cruise was organized by Nova Scotia Department of Energy in June-July 2016, using the Canadian Coast Guard Ship (CCGS) Hudson. This work was supported by Genome Canada, Genome Atlantic and Genome Alberta through a Genomic Applications Partnership Program (GAPP) award. Ship time funding and support was provided by the Nova Scotia Department of Energy and Mines, and Natural Resources Canada. Suitable footwear, gloves and equipments were used during the sampling process to minimize anthropogenic effects.
Disturbance	As disturbance was negligible due to very small volumes of collected samples, and thus no measure was taken to minimize this.

Reporting for specific materials, systems and methods

We require information from authors about some types of materials, experimental systems and methods used in many studies. Here, indicate whether each material, system or method listed is relevant to your study. If you are not sure if a list item applies to your research, read the appropriate section before selecting a response.

Materials & experimental systems

n/a	Involved in the study	
☒	☐	Antibodies
☒	☐	Eukaryotic cell lines
☒	☐	Palaeontology
☒	☐	Animals and other organisms
☒	☐	Human research participants
☒	☐	Clinical data

Methods

n/a	Involved in the study	
☒	☐	ChIP-seq
☒	☐	Flow cytometry
☒	☐	MRI-based neuroimaging
